# S155. SENSORY ATTENUATION DURING AUDITORY PROCESSING IN PARTICIPANTS AT CLINICAL-HIGH RISK FOR PSYCHOSIS: EVIDENCE FROM MAGNETOENCEPHALOGRAPHY

**DOI:** 10.1093/schbul/sby018.942

**Published:** 2018-04-01

**Authors:** Lingling Hua, Marc Recasens, Tineke Grent-’t-Jong, Emmi Mikanmaa, Hanna Thuné, Andrew Gumley, Matthis Schwannauer, Stephen Lawire, Ruchika Gajwan Gajwan, Joachim Gross, Peter Uhlhaas

**Affiliations:** 1 School of Psychology; 2 University of Glasgow; 3 University of Glasgow, Institute of Neuroscience and Psychology, Centre for Cognitive NeuroImaging, School of Psychology; 4 University of Edinburgh

## Abstract

**Background:**

The ability to predict the sensory feedback of self-generated stimuli against incoming sensory information is of importance to distinguish internal from external stimuli and is associated with sensory attenuation. Furthermore, it has been proposed that deficits in sensory attenuation could contribute to clinical symptoms of schizophrenia, including hallucinations and delusions, involving potential deficits in corollary discharge. The current study examined the hypothesis whether sensory attenuation is present in participants at clinical high–risk (CHR) for psychosis.

**Methods:**

Sixty-four CHR-participants and 32 healthy controls were presented with auditory stimuli during two experimental conditions: 1) In a passive condition, participants were presented with ripple sounds (40HZ with 2000ms duration, 83db) and responded to flat sounds (1000HZ with 2000ms duration,83db) with the right-index finger and 2) In an active condition, the ripple sounds were elicited by a button press with the right-index finger every 4s. MEG-data were acquired with a 248-magnetometers whole-head MEG system (MAGNES 3600 WH, 4-D Neuroimaging) at a sampling rate of 1017Hz. We focussed on the M100 response during the passive and active conditions at sensor- and source-level. A LCMV beamforming approach was employed for source reconstruction and virtual channels in primary auditory cortex and the left superior temporal cortex were used further analysis of sensory attenuation effects. Condition and group effects were tested with a cluster-based nonparametric test implemented in Fieldtrip with a window of interest for the M100 component between 120ms-150ms.

**Results:**

There was a significant decrease (P =0.009) in the amplitude of M100 component in the active vs. passive conditions across groups at both sensor- and source-level. Interaction-effects revealed that that sensory attenuation was significantly reduced in auditory cortices in the CHR group vs controls (P=0.032).

**Discussion:**

The current results highlight that sensory attenuation can be studied with ASSR-paradigms and that both primary auditory and superior temporal cortices underlie this effect. Moreover, our current findings suggest that sensory attenuation is impaired in CHR-participants, suggesting the possibility of impaired corollary discharge processes as a potential biomarker for the early diagnosis and detection of schizophrenia.

